# Reliable and transparent in-vehicle agents lead to higher behavioral trust in conditionally automated driving systems

**DOI:** 10.3389/fpsyg.2023.1121622

**Published:** 2023-05-18

**Authors:** Skye Taylor, Manhua Wang, Myounghoon Jeon

**Affiliations:** ^1^Mind Music Machine Lab, Grado Department of Industrial and Systems Engineering, Virginia Tech, Blacksburg, VA, United States; ^2^Link Lab, Department of Systems and Information Engineering, University of Virginia, Charlottesville, VA, United States

**Keywords:** trust, transparency, automated vehicles, in-vehicle agents, reliability

## Abstract

Trust is critical for human-automation collaboration, especially under safety-critical tasks such as driving. Providing explainable information on how the automation system reaches decisions and predictions can improve system transparency, which is believed to further facilitate driver trust and user evaluation of the automated vehicles. However, what the optimal level of transparency is and how the system communicates it to calibrate drivers’ trust and improve their driving performance remain uncertain. Such uncertainty becomes even more unpredictable given that the system reliability remains dynamic due to current technological limitations. To address this issue in conditionally automated vehicles, a total of 30 participants were recruited in a driving simulator study and assigned to either a low or a high system reliability condition. They experienced two driving scenarios accompanied by two types of in-vehicle agents delivering information with different transparency types: “what”-then-wait (on-demand) and “what + why” (proactive). The on-demand agent provided some information about the upcoming event and delivered more information if prompted by the driver, whereas the proactive agent provided all information at once. Results indicated that the on-demand agent was more habitable, or naturalistic, to drivers and was perceived with faster system response speed compared to the proactive agent. Drivers under the high-reliability condition complied with the takeover request (TOR) more (if the agent was on-demand) and had shorter takeover times (in both agent conditions) compared to those under the low-reliability condition. These findings inspire how the automation system can deliver information to improve system transparency while adapting to system reliability and user evaluation, which further contributes to driver trust calibration and performance correction in future automated vehicles.

## 1. Introduction

For years, psychologists, human factors specialists, engineers, and designers have been trying to find ways to improve user performance, acceptance, and trust with automated systems (Chen et al., 2018; Van de Merwe et al., 2022). With automated vehicles (AVs) just on the horizon of possibility for consumers, researchers have found that many factors influence human interactions with automation. The level of driving automation (LOA) (Cramer et al., 2008), presence and type of in-vehicle agent (IVA) (Dong et al., 2020), reliability of the system, and transparency of the system (Wright et al., 2020) are all some of the variables that influence user experience with AVs. The research surrounding reliability and transparency in automation has been thoroughly studied but resulted in mixed conclusions when it comes to the interactions between these two factors, in terms of trust and performance.

According to the Society of Automotive Engineers (SAE), fully automated vehicles are not currently available to consumers (SAE International, 2022). There are, however, vehicles on the roads that are considered conditionally automated. Our study explores human interaction with a conditionally automated vehicle, which requires drivers to take over control of the vehicle under certain circumstances, such as low visibility or technical failures (SAE International, 2022). The objective of this study is to investigate the effects of reliability and presentation of transparent system information in the context of conditionally automated driving. We are led by the following research questions:

RQ1: How do system reliability and information transparency influence drivers’ behavioral trust (i.e., a measure of trust based on quantifiable behavior, Adali et al., 2010; for the purposes of this study, measured as the number of takeover compliances) and performance when doing so?

RQ2: How do system reliability and information transparency affect drivers’ overall experience of the intelligent agent?

For the purposes of this study, the agent’s reliability and information presentation were manipulated to determine the effects of these variables on takeover, performance, and experience with the agent. Reliability was represented as the level of information accuracy. The highly reliable agent’s information was always accurate, and the unreliable agent shared some inaccurate information about the driving scenario. To elicit transparency from a robotic agent to a human user, the following “Robot-to-human” information factors are required: intention, task, analytics, and environment (Lyons, 2013). We designed two types of transparent agents, “what”-then-wait (on-demand) vs. “what + why” (proactive), each including all these factors and conveying the information *via* auditory message. The “what” information included factors regarding intention and environment, whereas the “why” information included factors regarding analytics and task. To examine the effects of information presentation, we manipulated how the “what” and “why” information pieces were conveyed to the user. The proactive agent provided both “what” and “why” information to the user in one instance. The on-demand agent only provided the “what” information unless the system was prompted to provide the “why” information by the user.

The unique contribution of the present study is twofold. First, the empirical data can help unpack the relationship between the system reliability and driver trust and show how the in-vehicle agent’s presentation of transparent information can mitigate its effects on driving behavior. Second, the results of the study can provide practical design guidelines for in-vehicle agents’ transparency with respect to system reliability.

### 1.1. Related work

#### 1.1.1. Trust

Trust is defined as “the attitude that an agent will help achieve an individual’s goals in a situation characterized by uncertainty and vulnerability” (Lee and See, 2004). This concept of trust is one of the main factors which influences people’s intentions to use AVs (Panagiotopoulos and Dimitrakopoulos, 2018). In the present study, we look at compliance as a measure of behavioral trust (Adali et al., 2010), meaning that the participants’ behavior indicates that they trust the agent/system. Although necessary for the adoption of AVs, trust is complex as it depends on many specific factors. One of the effective ways to improve driver trust and acceptance in AVs is to provide a tangible interface–such as a physical or virtual agent– to communicate system intentions and behaviors (Lee et al., 2019). In-vehicle agents (IVAs) have been proposed and evaluated in a wide context from manual driving (Johnsson et al., 2005; Nass et al., 2005; Maciej and Vollrath, 2009; Williams et al., 2013; Jeon et al., 2015), conditionally automated driving (Du et al., 2021; Mahajan et al., 2021; Wang et al., 2022), and fully autonomous driving (Large et al., 2019; Wang et al., 2021; Lee and Lee, 2022), to promote driver-automation interaction and improve driver performance.

Furthermore, although increased trust facilitates the reliance on automation, it can cause complacency and degrade monitoring performance of the driver (Parasuraman et al., 1993; Bailey and Scerbo, 2007), where monitoring is a critical user task in the context of conditional AVs because users are required to intervene and respond to system failures. Therefore, trust and automation have an “interdependent relationship” and must be properly calibrated to ensure the safe and effective use of automation (Lee and See, 2004). The influence of trust on performance has been studied across disciplines. In economics it has been shown to follow an inverted U-shape pattern with performance, where performance increases as trust increases until a certain point at which over-trust results in degraded performance (Villena et al., 2019). This has also been seen in the context of automation (Muir and Moray, 1996; Chancey et al., 2017). Two major factors in determining how to bolster and/or calibrate trust in AVs are examined in the present study: transparency and reliability.

#### 1.1.2. Reliability

Though ideal, AVs (along with the interfaces and in-vehicle agents integrated in the system to aid drivers) at their current technological evolution are not 100% reliable. Thus, an examination of how different levels of reliability impact drivers’ trust and performance is required. Reliability can be based on a machine’s ability to do what is asked of it when it is asked. When working with agents and assistants of automation, reliability can mean that the information they share is accurate. Decreased levels of system reliability have shown negative effects on users’ interactions with the system (Bliss and Acton, 2003). Also, previous research on automation reliability solidified that increased system reliability results in increased trust in and perceptions of the system (Kantowitz et al., 1997; Fox and Boehm-Davis, 1998; Bailey and Scerbo, 2007; Wright et al., 2020; Azevedo-Sa et al., 2021). However, in terms of takeover performance in automated vehicles, there are inconclusive results regarding the influence of the system reliability. For example, in an online study, Lanzer et al. (2021) showed that unreliable automation led to higher willingness to takeover. On the other hand, Zang and Jeon (2022) showed that a combination of high reliability and high transparency level led to higher maximum lateral acceleration in takeover, and a combination of low reliability and low transparency level also led to the similar outcome. Therefore, more research is still required on the effects of system reliability in automated vehicles.

Because trust is significantly increased when the reliability of an automated system is high, this results in complacency, which leads to degraded performance in monitoring the automation, despite better performance on secondary tasks (Bailey and Scerbo, 2007; Azevedo-Sa et al., 2021). Even when high reliability has shown improved human-automation performance, it was mainly at the 80% (Hanowski et al., 1994) and 100% (Mourant et al., 2015) levels. Our study will examine 100 and 67% reliability levels in terms of information accuracy.

#### 1.1.3. Transparency

The content and mode of the automation’s information presentation has a significant effect on trust (Lee and See, 2004). Trust in automated driving systems is specifically influenced by the level of information offered (Cramer et al., 2008). When using agents to assist human-interaction with automation, an understanding of the agents’ behavior is paramount for a beneficial interaction (Pokam et al., 2019). This level and mode of information sharing can be attributed to system transparency. Seong and Bisantz (2008) defined transparency as, “the extent to which the inner processes and decisions of an automation are made accessible.”

There are multiple benefits to transparent systems including improved performance, reduced human errors, and bolstered trust (Alonso and de La Puente, 2018). Many studies have exhibited increased trust in automation when the transparency of the system is increased (Entin and Entin, 1996; Cramer et al., 2008; Fan et al., 2008; Lyons, 2013; Mercado et al., 2016; Chen et al., 2018; Oliveira et al., 2020; Hartwich et al., 2021). However, when information about the system’s uncertainty level is presented, trust may decrease (Stowers et al., 2020).

Generally, increased transparency improved performance in human-agent teaming (Helldin et al., 2013; Koo et al., 2015; Bagheri et al., 2022; Shull et al., 2022). On the contrary, when working in time-sensitive contexts, such as driving a conditional AV, too much transparency can decrease performance due to the time needed for the system to present (and the user to process) the additional information (Stowers et al., 2020). Additionally, certain scenarios require less information so as not to overwhelm the operator (Bhaskara et al., 2020). Thus, the transparency of the system must be tailored to the available decision time (Entin and Entin, 1996).

How to deliver the correct type and amount of information to AV operators has been studied previously. Pokam et al. (2019) following Lyons’s principles of transparency, confirmed that some types of information are necessary for the safe operation of AVs. Namely, “information acquisition” (what the system understands about its environment) and the “action execution” (what the system plans to do in response) are essential for situation awareness (SA). Furthermore, according to the Situation Awareness-based Agent Transparency (SAT) model for automation, developed by Chen et al. (2018), SA and trust are greatly increased when the systems’ plan of action, reasoning process, and projections/expected outcomes are provided (SAT levels 1, 2, and 3, respectively) (Chen et al., 2018). Yet, when time sensitivity is a factor, the combination of all three of these information levels improves trust but decreases SA and performance, compared to when only SAT levels 1 and 3 are jointly provided (Bhaskara et al., 2020; van de Merwe et al., 2022).

#### 1.1.4. The interaction of reliability and transparency

If the transparency of a system is determined by the information provided to the user, then the reliability of that information is expected to influence the users’ performance and perceptions of that system. Nevertheless, only a few significant effects have been found between these two factors, and the results are mixed. Using an automation assistant in an automated target recognition task, Entin and Entin (1996) found that increased transparency resulted in the best performance when the reliability of the assistant was high, but the same level of transparency resulted in the worst performance when the system reliability was low, indicating complacency. However, a study on the reliability and transparency of a vehicle’s collision avoidance system found that increasing the amount of information *via* auditory messages negated the positive impact of a highly reliable system due to information overload (Mourant et al., 2015). This could be explained by Van de Merwe’s et al. (2022) findings that increased transparency has positive effects on SA up to a certain point, where too much information begins to diminish users’ SA and in turn, performance. However, Seong and Bisantz’s (2008) findings contradict this notion and claim, rather, that increased transparency mitigates decreased performance caused by low reliability. In terms of trust, increased transparency has shown to mitigate trust degradation caused by low reliability, but having little transparency in an unreliable system does not seem to influence trust (Kraus et al., 2020; Wright et al., 2020).

These contradictory findings suggest that further investigation is required to better understand the mechanisms between these variables. This study aims to explore the individual and interactive effects of reliability and presentation of transparent information on users’ trust, perceptions of in-vehicle agents, and performance in the context of a conditionally automated vehicle.

## 2. Method

### 2.1. Participants

A total of 30 participants (12 females) with normal vision or corrected-to-normal with glasses or contact lenses completed the study. The average age of participants was 24.57 (SD = 3.84). All participants held valid driver’s licenses with an average driving experience of 6.53 years (SD = 3.07). Participants’ driving frequency ranged from 1 time per week to 20 times, with an average of 6.16 times (SD = 3.89) per week. Participants provided informed consent and were compensated with a $10 cash payment for their participation.

### 2.2. Apparatus and stimulus

The Nervtech driving simulator (Nervtech™, Ljubljana, Slovenia) containing a car seat, steering wheel, and sport pedals was used for this study. Three large monitors provided a 120° horizontal view and consisted of three 48-inch HD TVs. The driving scenarios were developed using the SCANeR Studio, a driving simulation software program developed by the AV Simulation, run on a computer with an i7–8,086 K CPU and Nvidia GTX 1080 graphics card. Two driving scenarios were developed to simulate conditionally automated vehicles. Two driving scenarios consisted of both highway and urban roads, traffic signals and signs, and other road users such as pedestrians and other vehicles. All the traffic follows the speed limit, ranging from 50 km/h (31 mph) to 130 km/h (81 mph) depending on the traveling area (e.g., urban area, highway). All the vehicles were controlled by the traffic module within the SCANeR studio and traveled at the speed limit. The simulated subject vehicle had control of longitudinal and lateral motion for most of the time along a predefined route at the speed limit, while also handling limited road events such as following traffic lights and navigating intersections. When the automation system reached its limits due to suboptimal weather, or lighting conditions or surprising events, a speech takeover request (TOR) would be issued to notify the participants to take over control of the vehicle. The takeover time budget for the construction and severe weather events varied depending on the location and the event. These time budgets were tested in our pilot study to ensure that participants had sufficient time to react. If the participant decided to comply with the TOR, they could disengage the automated mode by either using a toggle attached to the steering wheel or pressing the brake. After the participant negotiated out the takeover event zone, the system prompted them to reengage the automated mode *via* a speech message, “Please reengage the auto-drive.” If participants decided not to comply with the TOR, the driving system would avoid collision with the obstacle in order to avoid distress from a crash in the simulator, which resulted in a shattered windshield and loud crashing noise. Each scenario consisted of three takeover events and two non-takeover events, lasting approximately 7 min. The routes differed in the specific events that occurred and the terrain covered across the two scenarios to mediate learning effects. Scenario 1 started at a stop light and the AV drove through a town for approximately 3.5 min before encountering the first TOR. The second scenario started on a highway and the AV encountered the first TOR after approximately 1.5 min. More details about the driving scenarios can be seen in Supplementary Tables S1, S2.

**Figure 1:**
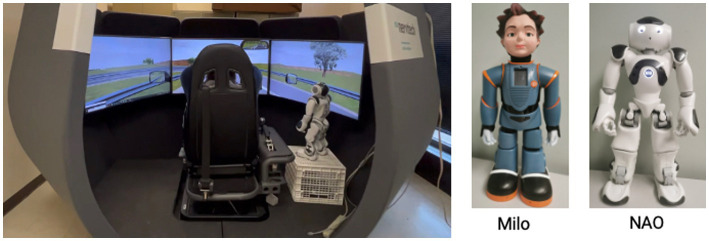
Experimental setup (left) and the two robots, Milo (middle), and Nao (right) used as embodied agents.

Two humanoid robots were used as in-vehicle agents (IVAs): Milo, by Robokind, and NAO, by SoftBank Robotics. Figure 1 shows the experimental set-up with NAO as the IVA. When Milo was used, the robot was placed at the same position as NAO. We decided to use embodied agents considering their potential benefits of enhancing the co-presence of the automation system and improving trust toward the system (Hock et al., 2016).

### 2.3. Experimental design

A 2 [Reliability: low vs. high] x 2 [Transparent information presentation: “what + why” (proactive) vs. “what”-then-wait (on-demand)] mixed factorial design was used, with reliability as a between-subjects variable and transparency as a within-subjects variable. Fifteen participants were assigned to the high-reliability condition, where 100% of the information presented was accurate. The remaining 15 participants experienced the low-reliability condition. Participants’ gender was balanced between two groups. In the low-reliability condition, the “unreliable agent” provided the driver with statements with only 67% accuracy for a certain event. Within the low-reliability condition, one of three information pieces was inaccurate. Supplementary Tables S1, S2 present the lists of agent intervention scripts. Within each reliability group, participants had two driving scenarios corresponding to two transparency conditions. They always experienced the proactive type of transparency condition first, meaning that the agent provided “what” was occurring in the scenario or “what” was the driver’s action commanded by the system, followed by the “why” information that clarified the reason behind it including system recognition and how it recognized. Then, participants experienced the on-demand type of transparency condition in their second drive, where they were only provided with “what” information, and the participants then had the opportunity to ask for more information up to two times. The sequence of transparency conditions was not balanced intentionally to show participants what types of information were available when the on-demand condition was implemented, but the matching between driving scenarios and transparency conditions was fully counterbalanced to minimize the order effects.

Agents with different transparency conditions were presented using two robots mentioned before. We intentionally utilized different robots to further distinguish two types of in-vehicle agents in terms of transparency condition. To prevent the appearance of the robot as a confounding variable, the orders for which each robot was assigned to the transparency condition were fully counterbalanced. Half of the participants had Milo as the proactive type of agent and NAO as the on-demand type agent; the remaining half of the participants experienced the opposite combination.

### 2.4. Dependent measures and data preprocessing

**Figure 2:**
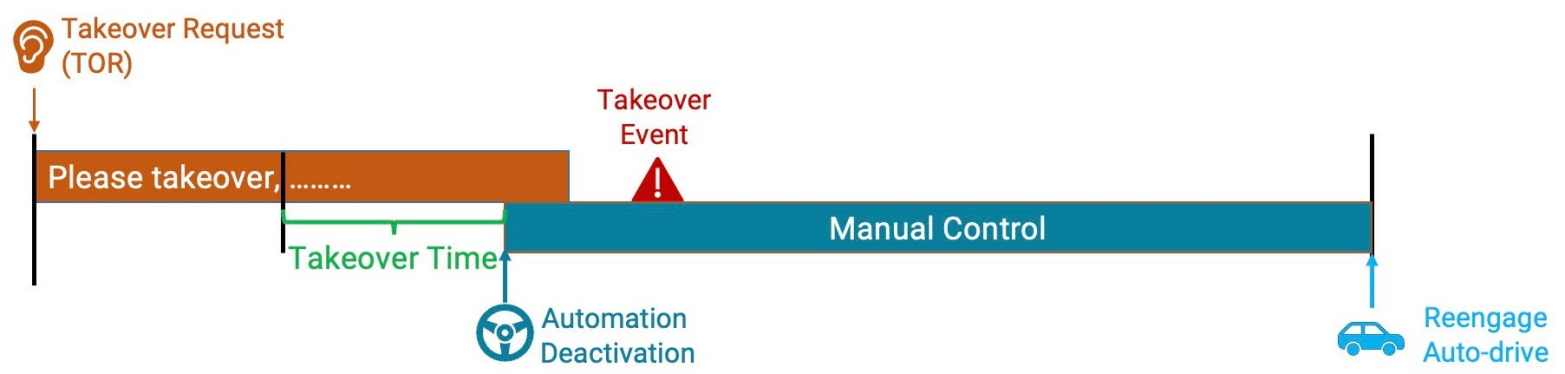
Takeover event process.

Both objective and subjective measures were used to evaluate the effects of reliability and transparency on drivers’ performance and perception. Objective measures mainly included takeover request (TOR) compliance (Zang and Jeon, 2022) and takeover performance. The number of times participants complied with the agents’ TOR was collected as an indicator for their decision-making. Takeover performance included takeover time and motor vehicle control. Takeover time was used as a temporal measure for takeover performance. The takeover time (in seconds) was calculated by the time difference between when the agent completed the takeover message segment (i.e., “please take over”) and when the auto-drive was disengaged if the participant chose to comply with the takeover request (Figure 2). Vehicle speed during the manual control period after takeover was used to assess the post-takeover maneuver that was applicable to all takeover events. The average, minimum, and maximum speeds (in m/s) were acquired to create a diverse profile for the longitudinal control. We also collected the maximum and standard deviation of the steering wheel angle (in rad) to assess participants’ lateral motor control after takeover. These two measures were analyzed separately for the lane-changing required event (i.e., construction) and non-lane-changing required events (i.e., fog, tunnel, and rain/fog).

Each participant completed four takeover events in each of the two driving scenarios. A total number of 231 data points resulting from 30 participants were collected for each takeover performance measure, excluding 9 instances where participants did not comply with the TORs. For each measure, values exceeding six standard deviations were reviewed and corrected if a programming error was detected, or removed if a true outlier was determined. The average values for the remaining data were calculated for each condition before conducting further analysis.

In addition to the takeover performance, subjective ratings of the two different agents were collected using previously validated questionnaires. The Subjective Assessment of Speech System Interfaces (SASSI) (Hone and Graham, 2000) scale was used to capture participants’ perception toward the in-vehicle agent as a speech user interface with a total of 34 items on six dimensions using a 7-point Likert scale: System Response Accuracy (9 items), Likability (9 items), Cognitive Demand (5 items), Annoyance (5 items), Habitability (4 items), and Speed (2 items). Participants’ trust toward the agent-automation system was evaluated by the Trust in Automation System scale, which included 12 items on 7-point Likert scales (Jian et al., 2000). Finally, participants’ perceived workload was measured *via* the NASA-Task Load Index (TLX) (Hart and Staveland, 1988). Participants’ preference toward the agent type was also collected (e.g., “Which voice agent do you prefer while driving?”). Their reasons behind the preferences were also collected.

### 2.5. Procedure

Upon arrival to the lab space, participants were asked to read and sign the informed consent that was approved by the university Institutional Review Board (IRB# 19–088). Before the formal testing, participants underwent a practice simulation following the Georgia Tech Simulator Sickness Screening Protocol (Gable and Walker, 2013), which also helped them get familiar with the simulation scenarios and the takeover process. Participants not suspected to have a simulation sickness tendency continued to complete the demographic questionnaire. Then, the participants were instructed that they were going to experience two driving scenarios in a conditionally automated vehicle, where they did not need to drive the entire time, but the in-vehicle agent would request them to take over control of the vehicle for certain events. Participants were also instructed that they did not have to comply with the takeover request; however, if they decided to take over the control, they must hand over the control upon system request. All participants went through the proactive type of transparency condition in their first drive, then completed the on-demand type transparency condition in their second drive. Additionally, participants were further instructed that the agent would only give them an initial piece of information about a certain event. Participants were informed that they could ask for more information by saying “more information,” and then the agent would provide more detail about the event (see Supplementary Tables S1, S2). After completing each drive, participants completed both the subjective questionnaires and NASA-TLX. Their preference toward the agent type was also collected. The study lasted approximately 60 min.

## 3. Results

Two-way repeated measures analysis of variance (ANOVA) was performed to understand differences in participants’ takeover performance and their subjective ratings. If any significant interaction effect was identified, a simple main effect analysis was conducted to further understand how reliability and transparency were interacted. For the number of compliances, independent-samples Manny Whitney U Tests was used. For the qualitative data collected for preference explanation, Affinity Diagrams (Holtzblatt and Beyer, 2017) was used to reveal common themes.

### 3.1. Reliability manipulation check

To validate that our manipulation on the reliability level was perceived accurately, we used one of the items in the System Response Accuracy subscale from SASSI– “The system was accurate” –as perceived reliability. Results from an independent samples t-test indicated that participants under the high reliability condition rated a higher degree of agreement on this statement (Mean = 5.73, SD = 0.82) compared to the ratings of those under the low reliability condition (Mean = 4.87, SD = 0.26): *t* (28) = 2.23, *p* = 0.017. Thus, our manipulation of system reliability was successful. The success of reliability manipulation can also be supported by participants’ comments at the conclusion of the study: the study:

*“There were number of wrong information, and I did not feel safe or reliable using the system.”* (P26 under low reliability condition)

### 3.2. Number of times more information was requested

**Figure 3:**
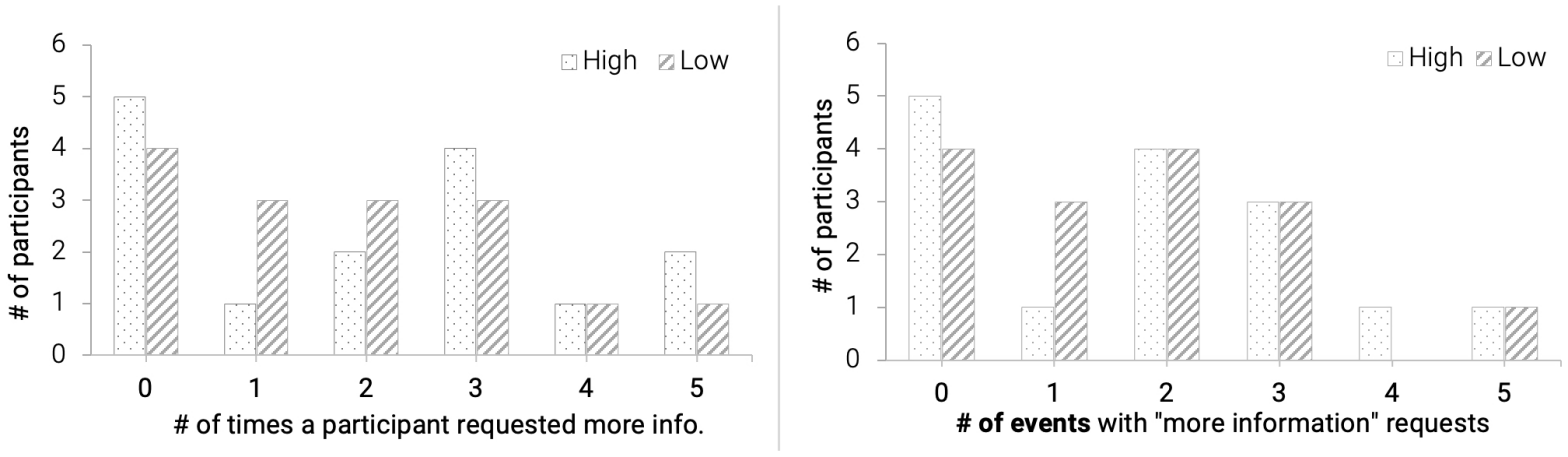
Frequency distribution for the number of times more information was requested.

We counted how many times participants asked for “more information” with the on-demand agent. For each event, participants could ask twice, resulting in 10 potential times for each participant. A total of 9 out of 30 participants did not request any more information (Figure 3, left). Results from a paired samples t-test indicated that the total number of times that a participant requested more information did not differ significantly between low reliability and high reliability groups: *t* (14) = 2.05, *p* = 0.67. We then looked at the number of events where participants requested further explanation. Only three participants asked for information for more than four events (Figure 3, right). The number of events in which more information was asked did not differ significantly between the two groups: *t* (14) = 2.05, *p* = 0.81. Thus, we anticipate that the information difference between participants who requested and did not request more information was small and would not have substantial influence on participants’ performance and subjective ratings.

### 3.3. Number of compliances with takeover request

**Figure 4:**
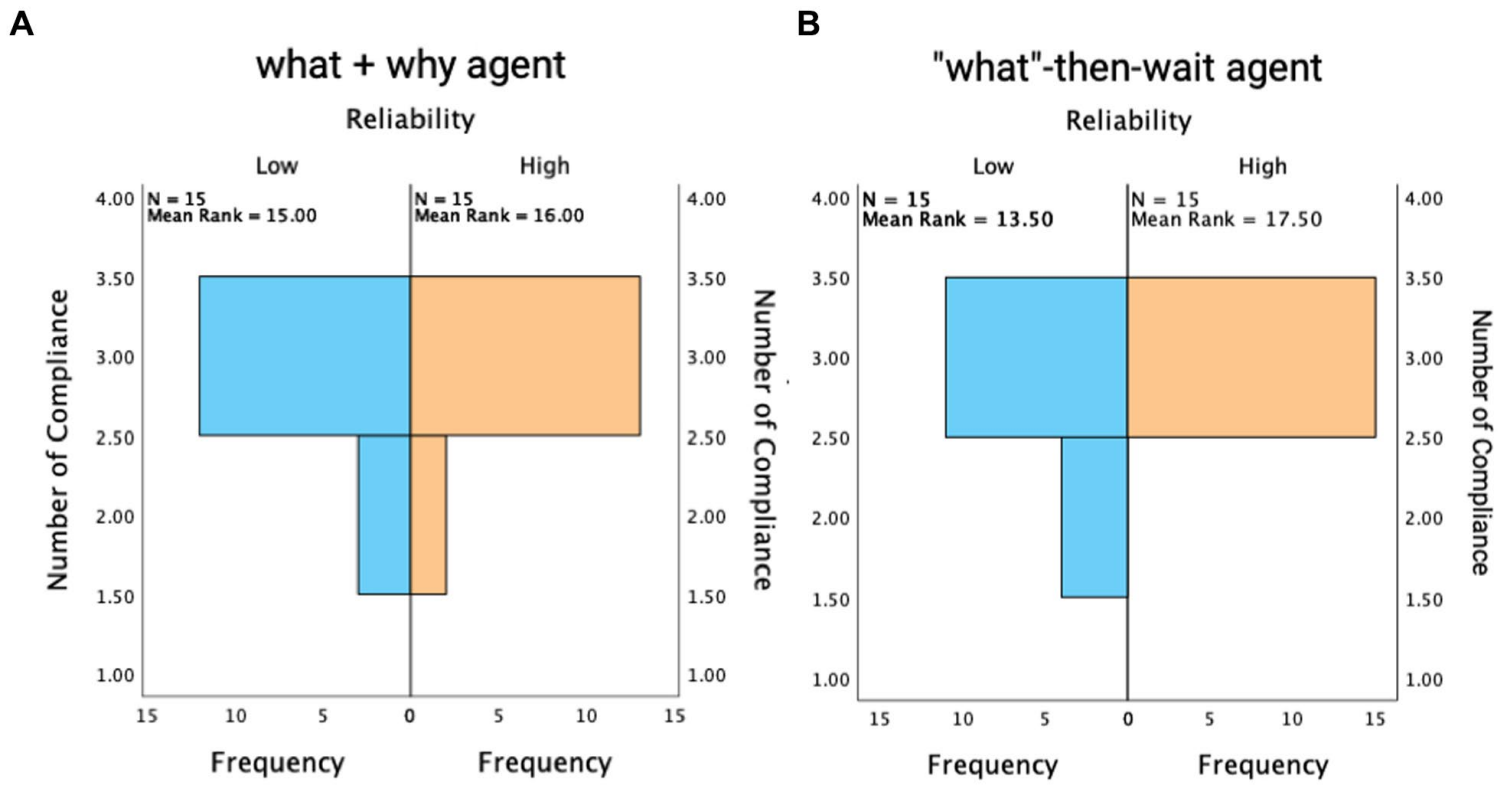
Number of compliances with TOR when accompanied by the what-and-why agent **(A)** and the “what”-then-wait agent **(B)**.

Independent-samples Manny Whitney U Tests were performed to understand participants’ compliances with the takeover request (TOR). For proactive agents who always presented information at once, there was no significant difference in the number of compliances between participants under low reliability and high reliability conditions, *U* = 105, *z* = −0.48, *p* = 0.63 (Figure 4A). However, with on-demand agents, participants under the low reliability condition complied less frequently than those under the high reliability condition, *U* = 82.5, *z* = −2.11, *p* = 0.035 (Figure 4B).

### 3.4. Takeover performance

Participants did not select different takeover methods (use the toggle vs. press the brake) across different conditions: $\chi^{2}$ (3) = 0.42, *p* = 0.92. Thus, the subsequent takeover performance analysis did not differentiate these two methods.

#### 3.4.1. Takeover time

**Figure 5:**
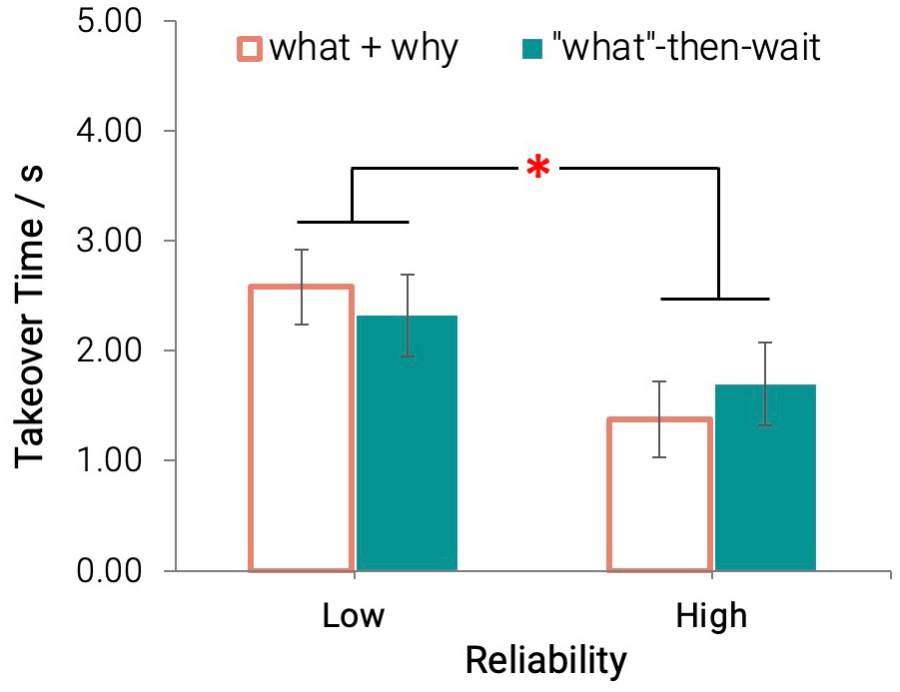
Takeover time across all conditions (*p < 0.05) [Error bars indicate standard errors].

Agent reliability had a significant main effect on drivers’ takeover time: *F* (1, 28) = 4.80, *p* = 0.037, $\eta_{p}^{2}$ = 0.15. Participants had a longer takeover time when the agents provided unreliable information (Mean = 2.45 s, SD = 1.10s) compared to the agents providing reliable information (Mean = 1.54 s, SD = 1.10s; Figure 5). Transparency did not show a significant main effect on takeover time: *F* (1, 28) = 0.01, *p* = 0.91.

#### 3.4.2. Post-takeover maneuver

**Figure 6:**
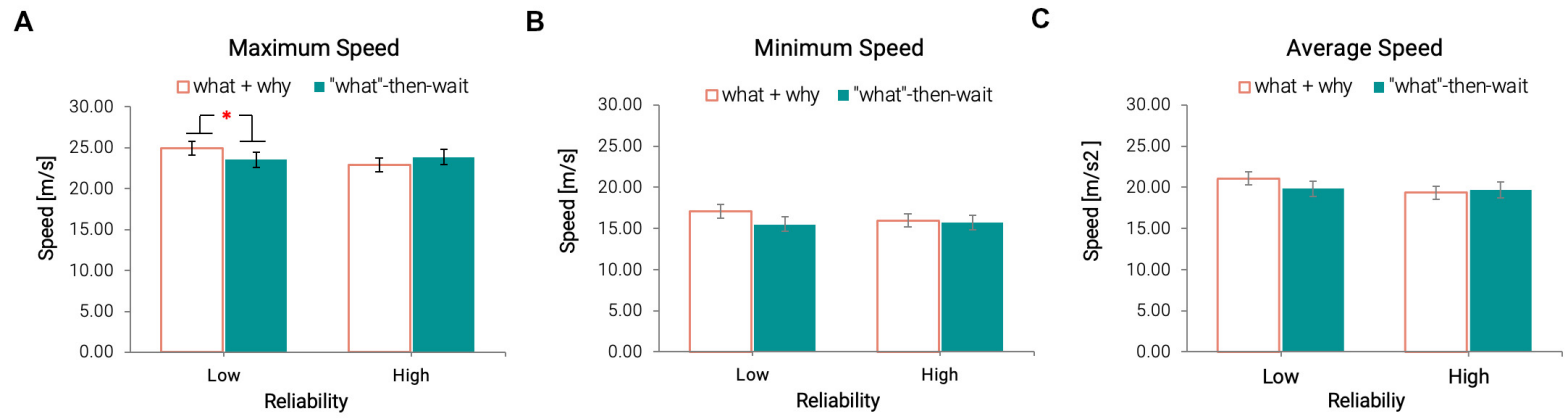
Maximum **(A)**, Minimum **(B)**, and Average **(C)** Speed across all conditions (*p < 0.05) [Error bars indicate standard errors].

The results indicated that reliability and transparency had a significant influence on speed-related measures. An interaction effect between reliability and transparency was found only on the maximum speed*: F* (1, 28) = 5.08, *p* = 0.032, $\eta_{p}^{2}$ = 0.15. Under the low reliability condition, participants drove faster when under the proactive condition (Mean = 24.93 m/s, SD = 3.74 m/s) compared to when they received only “what” information at first (Mean = 23.56 m/s, SD = 2.87 m/s; Figure 6A).

There was no main effect of either reliability or transparency on the minimum speed or the average speed after participants took over the control (Figures 6B,C).

As for the lateral motor control, for the lane-changing required event (i.e., construction), reliability did not have a significant effect on the maximum steering wheel angle (MAX_STW): *F* (1, 21) = 2.82, *p* = 0.108, or the standard deviation of the steering wheel angle (SD_STW): *F* (1, 25) = 1.62, *p* = 0.215. Transparency showed no significant main effect on these two measures either [MAX_STW: *F* (1, 21) = 0.99, *p* = 0.33, SD_STW: *F* (1, 25) = 0.065, *p* = 0.801].

For non-lane-changing required event (i.e., tunnel, fog, and jaywalker), reliability showed a tendency that participants had a slightly higher MAX_STW (Mean = 0.11 rad, SD = 0.04 rad) and SD_STW (Mean = 0.076 rad, SD = 0.01 rad) when the agents provided reliable information compared to the unreliable agents (MAX_SD: Mean = 0.077 rad, SD = 0.04 rad; SD_STW: Mean = 0.065 rad, SD = 0.01). But this tendency did not reach statistical significance [MAX_STW: *F* (1, 28) = 3.99, *p* = 0.056, $\eta_{p}^{2}$ = 0.13, and SD_STW: *F* (1, 28) = 3.30, *p* = 0.080, $\eta_{p}^{2}$ = 0.11]. Transparency did not have a significant main effect on either [MAX_STW: *F* (1, 28) = 0.52, *p* = 0.478, or SD_STW: *F* (1, 28) = 2.14, *p* = 0.155].

### 3.5. Subjective ratings on driver-agent interaction

**Figure 7:**
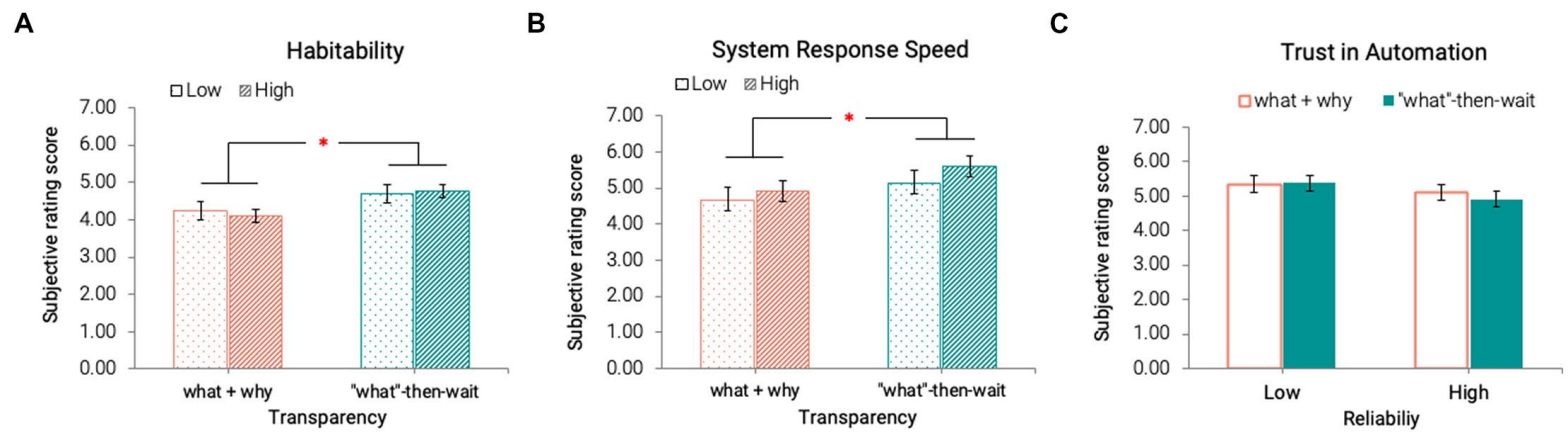
Habitability **(A)**, System Response Speed in SASSI **(B)**, and Trust in Automation **(C)** across all conditions (*p < 0.05) [Error bars indicate standard errors].

Results from repeated measures ANOVA revealed a significant main effect of transparency on the Habitability *F* (1, 28) = 7.68, *p* = 0.010, $\eta_{p}^{2}$ = 0.22 and (System Response) Speed *F* (1, 28) = 5.33, *p* = 0.029, $\eta_{p}^{2}$ = 0.16, subscales of SASSI. Agents with “what”-then-wait type of transparency were perceived as more habitable, meaning that the user’s conceptual understanding of the system was similar to that of the actual system (Hone and Graham, 2000; Figures 7A,B).

We did not observe any significant main effect of either reliability *F* (1, 28) = 1.98, *p* = 0.17, $\eta_{p}^{2}$ = 0.07 or transparency *F* (1, 28) = 0.14, *p* = 0.71, $\eta_{p}^{2}$ = 0.01 on perceived trust toward the agents (Figure 7C).

Considering that the appearance of the two agents used in our study was different, we also evaluated whether such a difference would affect driver trust. A paired samples t-test indicated that the agent’s appearance did not have a significant influence on driver trust, *t* (29) = 1.48, *p* = 0.14 (two-tailed).

### 3.6. Perceived workload

We only found a significant main effect of transparency on participants’ perceived effort: *F* (1, 28) = 4.08, *p* = 0.05, $\eta_{p}^{2}$ = 0.13. Participants perceived that they had to work more to accomplish their levels of performance when the agent only presented “what” information first. No significant differences were found on other dimensions or overall workload. See Supplementary Table S3 for all NASA-TLX ratings.

### 3.7. Agent preference

**Figure 8:**
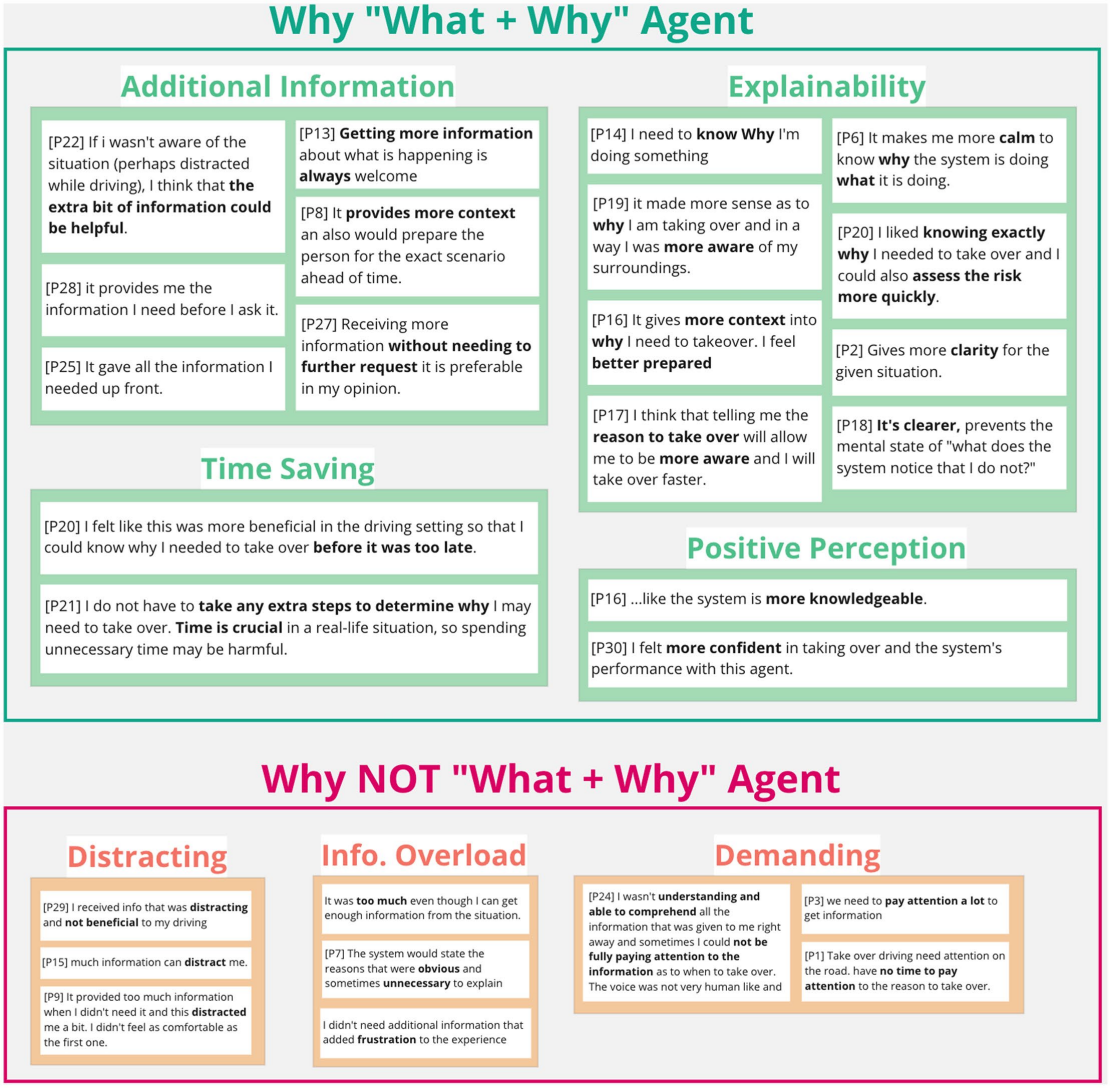
Participants’ comments on their like and dislike of “what + why” agent.

Eighteen out of thirty participants (60%) favored the agents that proactively provided “what + why” information at once. The affinity diagrams organizing participants’ explanations indicated that such preference came from the additional information provided by the proactive agent, the explanation on the action required, the time saved from reducing extra information acquisition, the resulting positive perception toward the system (e.g., “more knowledgeable”), and their own behaviors (e.g., “I felt more confident”; Figure 8, top). However, not all participants found that the additional information was helpful but rather distracting. The additional information can be overloading and demanding, especially when it was considered “unnecessary” (Figure 8, bottom).

**Figure 9:**
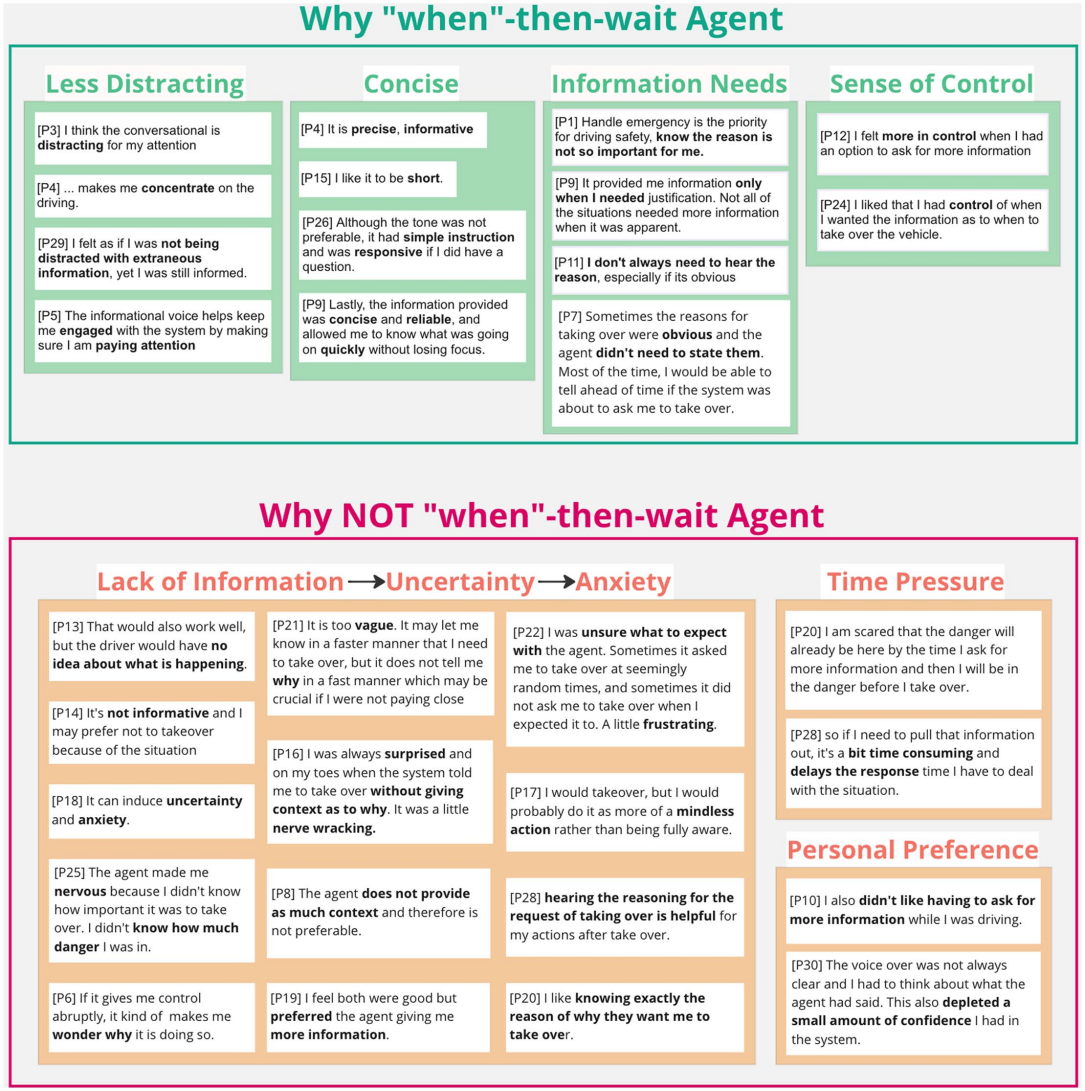
Reasons behind likes and dislikes toward the “what”-then-wait agent.

The remaining 12 participants (40%) preferred the agents that only provided “what” information at first. In contrast to the proactive agent, the on-demand agent was less distracting and concise (Figure 9, top). The extra information request created a sense of control and fulfilled the information needs as necessary (Figure 9, top). However, most participants perceived that the lack of information yielded great uncertainty, thus, generated anxiety (Figure 9, bottom). Participants also argued that the extra step of pulling information can be dangerous under time pressure during the takeover events.

## 4. Discussion

The objective of the present study was to investigate the effects of embodied in-vehicle agents’ reliability and information transparency on users’ takeover performance and trust in conditionally automated vehicle systems. Although we did not identify any changes in users’ subjective trust, we observed different levels of behavioral trust (i.e., the number of compliances) in response to system reliability and transparency. Our results indicate that although the system reliability has the decisive impact on the takeover time, the influence of system reliability on drivers’ takeover performance also largely depends on the system transparency. Additionally, the system transparency has greater impact on the driver perception toward the in-vehicle agents.

### 4.1. System reliability and transparency impact behavioral trust (RQ1)

Our findings indicated that the effect of reliability on drivers’ willingness to comply with the takeover requests (TORs) might depend on the agent type. When the agent proactively provided “what + why” information at once, drivers did not exhibit different takeover behaviors in terms of compliance. However, when the agent provided information on-demand, drivers complied less with the TORs if the system reliability was low with 67% information accuracy compared to when it was high with 100% accuracy. Compliance with a system serves as an indirect measure of user trust in automated systems (Chancey et al., 2017): higher trust is associated with a greater tendency to comply (Fox and Boehm-Davis, 1998). The compliance pattern found in our study indicated that an automation system that provided additional explanations (i.e., what + why) hindered drivers’ ability to build appropriate trust toward systems with different levels of reliability. Even when the message included inaccurate information in the low reliability condition, drivers still developed the same high level of trust toward the unreliable agents as they developed their trust toward the reliable agents (Figure 4). Forming such high-level trust not matching with the system’s low capability can lead to overreliance on an automation system, which further predicts potential system misuse–overlooking the automation limitations and relying on it inappropriately (Lee and See, 2004). In case of uncertainties, limiting the information provided might be a solution to prevent over trust. Our findings indicated that without such explanations, drivers were less likely to comply with the system because they experienced difficulties forming an understanding of the system functions and thus, challenged the necessities of takeover.

**Figure 10:**
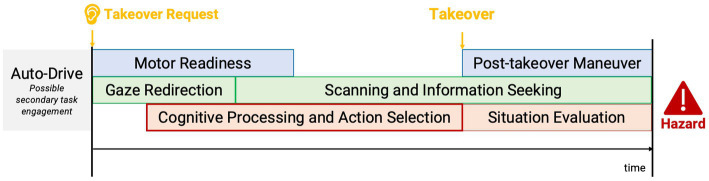
A conceptual model of takeover process after a speech takeover request (adapted from Zeeb et al., 2015; McDonald et al., 2019).

Our findings indicate that if drivers decided to take over control, system reliability would play a critical role in drivers’ takeover time. With a low reliable system providing only 67% accurate information, drivers had a significantly longer takeover time compared to their reaction with a highly reliable system providing 100% accurate information. Figure 10 depicts a conceptual model of the takeover process adapted from Zeeb et al. (2015) and McDonald et al. (2019). In safety-critical takeover events presented in our study, a faster takeover time ensured the driver adequate time to be ready to regain control of the vehicle and develop appropriate strategies to negotiate the hazardous situations. However, with inaccurate information provided from an unreliable system, drivers had longer latency in the cognitive processing and action selection stage, where they had to reevaluate the information after redirecting their gaze on the road and searching for confirmative information. Then, they were able to take actions accordingly. Such an additional cognitive process resulted in a delayed takeover action. Existing evidence from eye-tracking research also indicated that people spent more time on low reliability automation systems and visited them more frequently in a monitoring task (Lu and Sarter, 2019).

The effects of system reliability on post-takeover maneuvers interact with the system transparency. When the system reliability was low, drivers drove faster after takeover when the system provided “what + why” information all in once compared to when the system provided only “what” information at first. As we mentioned above, drivers built up confidence in their actions with an explainable system. However, the unreliable system delayed their takeover action and left them less time to strategize actions. Consequently, confident drivers might exhibit risky driving behaviors–a higher speed in this case–to negotiate the hazards within limited time. Comparably, without further explanation, a less confident driver informed by an unreliable system might act with caution and drive conservatively.

### 4.2. Information transparency largely impacts driver experience (RQ2)

Although a smaller number of participants preferred the “what”-then-wait type agent, findings in subjective ratings indicated that drivers rated this type of agent with higher Habitability and (System Response) Speed scores. The habitability subscale measures the extent to which the driver knows what to do to interact with the system (Hone and Graham, 2000). Because drivers were informed that they were allowed to request more information by saying the requesting phrase, they were more aware of their options compared to when interacting with the proactive agent as receptors. The existing evidence has supported that habitability is a key factor in speech system usability (Hone and Baber, 2001). Thus, in-vehicle intelligent agents as a speech user interface should also be designed in a habitable way.

The speed subscale measures how quickly the system responds to user inputs, measured by two statements: (1) “The interaction with the system is fast” and (2) “The system responds too slowly.” Conciseness was one of the advantages of the “what”-then-wait type agent, who provided the information segment in small pieces and allowed additional information per request. Such conciseness might yield a feeling of faster system response speed. On the contrary, the proactive agent provided all information at once. The syntactic and semantic complexity also increased as the information richness expanded. Some drivers struggled to understand the system output when the information got complicated, and the processing time was prolonged as a consequence (Hone and Graham, 2000). Participants commented that some of the additional information were unnecessary and already obvious to them. Thus, the information value–the quantifiable measure of uncertainty reduction (Howard, 1966)–is critical to promote user experience and trust in the system, as long as the information added does not increase the complexity of the message.

We identified an increased effort experienced by participants when using a “what”-then-wait type agent. Based on participants’ comments, the limited information provided by this type of agent created great uncertainty. Such uncertainty demanded drivers to exert extra mental effort to seek useful information from the environment before taking effective maneuvers. All these cognitive activities happen under time pressure. A trustable in-vehicle agent should demonstrate an attitude to help achieve drivers’ goals in an uncertain and vulnerable situation (Lee and See, 2004). Thus, the “what”-then-wait type agent was not able to facilitate trust.

Finally, in terms of agent preference, the on-demand agent was less preferred compared to the proactive agent. This finding aligns with the previous research that “what”-only explanation on a vehicle’s action had the lowest acceptance (Koo et al., 2015). The proactive agent who provided “what + why” information at once was slightly preferred in our study. Existing evidence has supported the benefits of having such an agent in promoting trust (Forster et al., 2017; Ha et al., 2020) and acceptance (Koo et al., 2015; Forster et al., 2017; Naujoks et al., 2017) of automated vehicles (AVs). The “what + why” explanation also made the environmental information more accessible for inattentive drivers. We also found mixed feelings toward the proactive agent, especially when the prompts were too long to be digested. Previous research studies have also found that what + why explanation can increase annoyance compared to the single piece explanation (i.e., “what” only or “why” only; Koo et al., 2015, 2016). Such negative feelings might come from the compromised explanation goodness and explanation satisfaction when presenting the information. Hoffman et al. (2018) have pointed out that explanation is not the cluster of statements, but depends on the user needs, existing knowledge, and especially, goals. Because some information provided by the proactive agent overlapped with drivers’ existing knowledge, it was perceived as unnecessary and thus, may have hurt the explanation goodness and explanation satisfaction.

## 5. Limitations and future work

This study provides a deeper understanding of how drivers perceive an automated vehicle and its agent depending on specific combinations of multiple factors (i.e., reliability and transparency of the intelligent agent), but we acknowledge several limitations of this work that can be further examined. First, the order of the transparency conditions was not counterbalanced in such a way that participants could learn the information available for them to request thereafter. Although different humanoid robots were matched and counterbalanced with different transparency conditions that could create a perception of different agents presented, participants’ experience with the automation system can be affected by this fixed sequence. In the future, researchers with a similar dilemma can educate participants on the available information provided by the system and allow them to experience several examples using sample events that are different from the experimental condition. Second, the manipulation of the reliability conditions was slightly compromised with the “what”-then-wait type agent. If the participant did not ask for more information, they lost the chance to receive as much false information, depriving the agent’s opportunity to be interpreted as reliable or not. However, since we presented the proactive agent first, participants may have had an anchored trust level to low reliability or to high reliability. This anchoring effect impacted their subsequent trust in the second drive and set the initial trust level similar to the first one. Thus, we believe the reliability manipulation was still valid in their second drives. However, we also acknowledge that there might be better approaches to examine our manipulation check on reliability, such as directly collecting participants’ perceived system reliability level as low, medium, and high. Additionally, empirical research has identified several major factors that influence TOR performance. Mainly, repeated exposure to the takeover task could improve takeover time (McDonald et al., 2019). Participants practiced the takeover task during the training scenario, and the order of conditions was counterbalanced to minimize the influence of order effects and enhance the study’s internal validity. However, the familiarity could have manifested as the drivers experienced multiple opportunities to conduct the takeover action.

Despite these limitations, the findings from our study can inspire future investigation on how different settings of reliability and transparency can further impact driver trust and their performance. With limited technology, uncertainty in system information cannot be eliminated in the near future. Findings from the existing literature converge toward the idea that users have some tolerance for information accuracy (Kantowitz et al., 1997; Fox and Boehm-Davis, 1998), but the fine point of accuracy tolerance has not reached an agreement (around 60–70% accuracy). A deeper look into the threshold of an acceptable accuracy level is needed to accommodate the technology limitations. In addition, our results indicated that “what + why” type information can increase user trust toward the system and improve their confidence in taking actions. However, such elevated trust might have unintended consequences if the information provided was unreliable. With the foreseeable system unreliability, always providing explanations might not be beneficial all the time. Future research can be done to explore an appropriate approach to deliver only relatively reliable information or convey unreliable information in a way that allows people to be aware of its inaccuracy and take that into consideration. For example, an adaptive agent can be developed to adjust the information transparency in response to different events in terms of urgencies, and also strategize according to the reliability level. In a future study, it would also be beneficial to include a baseline level of transparency to evaluate whether information about the driving scenario is required at all during a TOR.

## 6. Conclusion

In the present study we evaluated the effects of reliability and transparency of in-vehicle agents (IVAs) on drivers’ takeover performance, their trust, and their perception toward the automation system in conditionally automated vehicles. Our findings indicated that high system reliability in general increased drivers’ takeover request compliance rate and shortened the takeover time, which might suggest an increase in drivers’ trust. However, the increased trust and user perception by the proactive agent can have unintended consequences that lead to risky driving behaviors when the system is unreliable. This interaction effect between system reliability and information transparency sheds light on the importance of agent adaptability according to user contexts and system limitations. Based on our findings, we have the following design recommendations:

- Information provided by IVAs should be set to an appropriate reliability level to accommodate for the system uncertainty while also promoting user trust and encouraging safer driving behaviors.
- Information transparency should be adjusted carefully according to system reliability to avoid false trust and confidence, and the subsequent influence on driving behaviors.
- IVAs should be designed in a habitable way to assist drivers in forming a correct conceptual model of interacting with the automation system, which can further improve their performance and experience.

## Data availability statement

The raw data supporting the conclusions of this article will be available upon request.

## Ethics statement

The studies involving human participants were reviewed and approved by Virginia Tech Institutional Review Board. The participants provided their written informed consent to participate in this study.

## Author contributions

ST: collecting of the data, data curation, and writing the draft. MW: theoretical foundations, modifying driving scenarios, data analysis, and writing the draft. MJ: funding acquisition, conceptualization, research design, reviewing and editing, and supervision. All authors contributed to the article and approved the submitted version.

## Funding

This project was funded by the Northrop Grumman Research Experience Grant Award.

## Conflict of interest

The authors declare that the research was conducted in the absence of any commercial or financial relationships that could be construed as a potential conflict of interest.

## Publisher’s note

All claims expressed in this article are solely those of the authors and do not necessarily represent those of their affiliated organizations, or those of the publisher, the editors and the reviewers. Any product that may be evaluated in this article, or claim that may be made by its manufacturer, is not guaranteed or endorsed by the publisher.
