# Supplementary material for: Reliable and transparent in-vehicle agents lead to higher behavioral trust in conditionally automated driving systems
Source: Front Psychol. 2023 May 18;14:1121622. doi: 10.3389/fpsyg.2023.1121622 (PMC10232983; doi:10.3389/fpsyg.2023.1121622)
Supplement: Supplementary file 1 [file Table_1.docx]

**Supplementary Table 1. Scenario 1 intervention scripts**

| Event | Proactive | On-demand |
| --- | --- | --- |
|  | **[Reliable /** *Unreliable*] | **[Reliable /** *Unreliable*] |
| Construction Site | Please take over. The vehicle's front cameras detect an obstacle [**around a quarter mile** / *3 miles*] ahead | 1. Please take over  2. Obstacle [**around a quarter mile** / *3 miles*] ahead  3. Detected by front cameras |
| Car Swerves | The car in front of you is expected to swerve into your lane in [**1000 feet** / *2 miles*] based on the system's prediction program. | 1. The car in front of you is expected to swerve into your lane  2. It is expected to swerve in [**1000 feet** / *2 miles*]  3. Detected in the system’s prediction program |
| Decision Error | Please take over. There is an error in the system's decision-making code.    *…Never mind* | 1. Please take over  2. System Error  3. Detected in system's decision code.  4. *Never mind (given regardless of whether the participant asks for more info)* |
| Jaywalker | The vehicle's front right sensors detect a [**pedestrian ahead who is walking into the street** / *large animal crossing the road ahead*]  Based on their trajectory, the vehicle will brake and move to the left lane. | 1. [**Pedestrian on road** / *Animal crossing road*] ahead  2. Based on the pedestrian's trajectory, the vehicle will brake and move to the left lane  3. Detected by front right sensors |
| Fog | Please take over. The vehicle's light sensors detect heavy [**fog** / *rain*] ahead. | 1. Please take over  2. Heavy [**fog** / *rain*] ahead  3. Detected by vehicle's light sensors |

Note: **Bolded** parts were presented in the high-reliability condition. *Italic* parts were presented in the low-reliability condition
