# Supplementary material for: Reliable and transparent in-vehicle agents lead to higher behavioral trust in conditionally automated driving systems
Source: Front Psychol. 2023 May 18;14:1121622. doi: 10.3389/fpsyg.2023.1121622 (PMC10232983; doi:10.3389/fpsyg.2023.1121622)
Supplement: Supplementary file 2 [file Table_2.docx]

**Supplementary Table 2. Scenario 2 intervention scripts.**

| Event | Proactive | On-demand |
| --- | --- | --- |
|  | **[Reliable /** *Unreliable*] | **[Reliable /** *Unreliable*] |
| Construction Site | Please take over. The vehicle's front cameras detect an obstacle [**700 feet** / 2 *miles*] ahead | 1. Please take over  2. Obstacle around [**700 feet** / 2 *miles*] ahead  3. Detected by front cameras |
| Car Swerves | The vehicle to your [**left** / *right*] is expected to swerve into your lane based on the system's decision model. | 1. Vehicle expected to move into your lane  2. The vehicle is positioned on your [**left** / *right*]  3. Detected in system's decision model |
| Sensor Malfunction | Please take over. The front right sensor is malfunctioning based on the test code.    *…Never mind* | 1. Please take over  2. Sensor malfunction  3. Detected in the system's decision code.  4. *Never mind (given regardless of whether the participant asks for more info)* |
| Cow in road | The vehicle's front right sensors detect a [**large animal** */ child*] crossing the road ahead.  Based on their trajectory the vehicle will brake and move to the left lane. | 1. [**large animal /** *child*] in the road ahead  2. Based on the pedestrian's trajectory, the vehicle will brake and move to the left lane  3. Detected by front right sensors |
| Rain | Please take over. The vehicle's moisture sensors detect heavy [**rain** / *fog*] ahead. | 1. Please take over  2. Heavy [**rain** / *fog*] ahead  3. Detected by vehicle's moisture sensors |

Note: **Bolded** parts were presented in the high-reliability condition. *Italic* parts were presented in the low-reliability condition
