# Supplementary material for: Reliable and transparent in-vehicle agents lead to higher behavioral trust in conditionally automated driving systems
Source: Front Psychol. 2023 May 18;14:1121622. doi: 10.3389/fpsyg.2023.1121622 (PMC10232983; doi:10.3389/fpsyg.2023.1121622)
Supplement: Supplementary file 3 [file Table_3.docx]

Supplementary Table 3. NASA-TLX ratings across all conditions [Mean (SD)]

|  | **Reliability** | **Transparency** | |
| --- | --- | --- | --- |
|  |  | **proactive** | **on-demand** |
| **Mental Demand** | Low | 34.00 (19.48) | 35.67 (27.38) |
|  | High | 35.67 (21.37) | 39. 67 (25.53) |
| **Physical Demand** | Low | 18.67 (10.60) | 17.3 (9.80) |
|  | High | 23.00 (16.56) | 25.33 (17.47) |
| **Temporal Demand** | Low | 38.00 (22.98) | 39.00 (29.04) |
|  | High | 37.33 (23.44) | 37.67 (23.97) |
| **Performance** | Low | 29.33 (16.02) | 30.33 (22.79) |
|  | High | 32.00 (16.67) | 36.33 (27.61) |
| **Effort*** | Low | 29.00 (12.85) | 31.33 (22.95) |
|  | High | 24.67 (16.42) | 35.00 (22.99) |
| **Frustration** | Low | 20.33 (13.16) | 28.00 (22.50) |
|  | High | 23.00 (19.35) | 24.00 (23.54) |
| **Overall Workload** | Low | 32.55 (14.09) | 34.04 (23.36) |
|  | High | 32.33 (15.26) | 37.36 (22.73) |
